# Causal relationship between metabolites and embolic stroke: based on Mendelian randomization and metabolomics

**DOI:** 10.3389/fneur.2024.1460852

**Published:** 2024-10-11

**Authors:** Ying Hang, Zanhao Chen, Jiayi Ren, Yu Wang, Kangle Zhu, Qianhong Zhu

**Affiliations:** ^1^Radiography Center - Interventional Catheter Room, Yixing People’s Hospital, Yixing, China; ^2^Department of Medicine, Xinglin College, Nantong University, Nantong, China; ^3^Nanjing Drum Tower Hospital Clinical College of Nanjing Medical University, Nanjing, China; ^4^Department of Emergency Medicine, Yixing People’s Hospital, Yixing, China

**Keywords:** metabolite, Mendelian randomization, embolic stroke of undetermined source, genome-wide association study, inverse variance-weighted

## Abstract

**Purpose:**

This research employed Mendelian randomization (MR) methods to explore whether metabolites are causally associated with embolic stroke of undetermined source (ESUS).

**Methods:**

Genome-Wide Association Study (GWAS) data regarding metabolites and ESUS were downloaded from the database. Metabolites were employed as exposure factors, ESUS served as the outcome variable, and single nucleotide polymorphisms (SNPs) exhibiting significant association with ESUS were chosen as instrumental variables. The causal association between exposure factor metabolites and the outcome variable ESUS was assessed using two methods: MR-Egger regression and inverse variance-weighted (IVW) analysis.

**Results:**

A causal relationship was observed between X-11593--O-methylascorbate* and ESUS, indicating a protective factor. Moreover, a causal relationship was identified between cholesterol esters in large very-low-density lipoprotein (VLDL), cholesterol esters in medium low-density lipoprotein (LDL), concentration of medium LDL particles, phospholipids in medium LDL, phenylalanine, total cholesterol in small LDL, total lipids in medium LDL and ESUS, representing risk factor. Funnel plots exhibited a symmetrical distribution of SNPs, while pleiotropic tests (*p* > 0.05) and leave-one-out tests indicated that the results were relatively stable.

**Conclusion:**

Metabolites are causally associated with ESUS. LDL and VLDL-related metabolites are identified as risk factors for ESUS.

## 1 Introduction

Stroke is a serious disease marked by high morbidity, recurrence, disability, and mortality rates. It poses significant health risks and economic burdens. The main pathological types are ischemic and hemorrhagic stroke, with embolic stroke of undetermined source (ESUS) accounting for about 17% of ischemic stroke patients (1). ESUS refers to non-lacunar ischemic stroke where intracranial or extracranial vascular stenosis and cardiogenic emboli are excluded (2). Currently, effective treatment options for ESUS remain limited. Therefore, understanding the etiology and risk factors of ESUS and identifying potential diagnostic treatments are crucial for public health (3).

Metabolites are intermediates or end products of metabolic reactions and their levels can be impacted by numerous factors, including diet, genetics, gut microbiota, lifestyle, and disease (4). Through metabolite analysis, the mechanisms of disease development can be understood, targets for disease intervention can be sought, disease risk can be mitigated, and stratified prevention and management can be achieved (5). Currently, the causal function of metabolites in disease etiology has been demonstrated, providing new ideas for diagnosis and treatment. Additionally, many metabolites exhibit high heritability, making human genetics a method for assessing the impact of metabolites on disease outcomes.

Building on this understanding, Mendelian Randomization (MR), a causal inference method, has recently gained widespread usage in the field of epidemiology. This approach relies on genome-wide association study (GWAS) data, utilizing genetic variants as instrumental variables (IVs) in order to examine the impact of exposure on disease outcomes (6). Due to the random assignment of genetic variation at conception, this approach can break the confounding of most risk factors and reduce the tendency of confounding to bias the results, thus achieving the effect of simulating randomized controlled trials. In this research, MR analysis methods utilizing GWAS data were employed for the investigation of the causal association between metabolites and ESUS. The aim was to present novel ideas and directions for the future diagnosis and treatment of ESUS.

## 2 Methodology

### 2.1 Data selection

In this study, metabolite-related GWAS data were used as exposure factors. Single nucleotide polymorphisms (SNPs) exhibiting significant association with ESUS were employed as IVs, while ESUS-associated data were used as outcome variables.

### 2.2 Data sources

Metabolite-related data and GWAS data for ESUS in this study were derived from databases encompassing https://gwas.mrcieu.ac.uk/, https://www.ebi.ac.uk/gwas/, and https://www.finngen.fi/en. Metabolite-related GWAS data utilized in this research mainly included met-a-536, met-c-881, met-c-905, met-c-906, met-c-907, met-c-908, met-c-919, and met-c-924. GWAS data for ESUS was I9_STR_EMBOLIC. As this study relied on publicly available data, ethical approval or consent was not required.

### 2.3 Relevance analysis

MR Assay meets the following three hypothetical premiums:

① Relevance hypothesis: significant association between IVs and metabolites (exposure factors);

② Independence hypothesis: lack of association between IVs and ESUS (outcome variable) and other confounding factors;

③ Exclusivity hypothesis: IVs can only influence ESUS (outcome variable) by affecting metabolites (exposure factors) and cannot influence ESUS through other pathways (7).

SNPs strongly associated with exposure factors were selected as IVs and filtered at the condition of *p* < 5e-08.

### 2.4 Linkage disequilibrium

Linkage disequilibrium (LD) is the phenomenon where genetic variants located close to each other on a chromosome tend to be inherited together more often than would be expected by chance. This means that the likelihood of specific alleles (gene variants) from two or more loci appearing together on a single chromosome is greater than if the alleles were distributed randomly (8). To mitigate potential bias from LD, the screening criteria for single nucleotide polymorphisms (SNPs) are outlined below:

1. Kb > 10,000;
2. *r*^2^ < 0.001.

Here, Kb refers to the range of regions in LD. *r*^2^ is evaluated between 0 and 1, where *r*^2^ = 1 denotes a complete LD relationship between two SNPs, while *r*^2^ = 0 suggests a complete linkage equilibrium relationship between two SNPs, indicating the random assignment of these two SNPs (9).

### 2.5 Removing weak tool variables

To avoid bias for weak IVs, the F-statistic was utilized to determine their strength. An IV with an *F*-value <10 was defined as weak, while an F-value >10 was considered a non-weak IV (10). The strength of selected individual tool variables was evaluated by formula ( $F=\frac{N-K-1}{K}\times \frac{R^{2}}{1-R^{2}}$ ), with N representing the total sample size of GWAS data exposed, K representing the number of screened IVs, and R^2^ representing the proportion of exposures explained by IV. *R*^2^ is calculated as *R*^2^ = 2 × (1-MAF) × MAF × (*β*/SD)^2^, where MAF represents the minor allele frequency, β indicates the effect size of the allele, and SD denotes the standard deviation (11).

### 2.6 Mendelian randomization analysis

MR-Egger regression and inverse variance-weighted (IVW) analysis were employed to reveal the causal association between exposure factor metabolites and outcome variable ESUS. IVW was utilized as the main method to evaluate the reliability of the findings, defining *p* < 0.05 as a positive result (12). The R package “TwoSampleMR” was utilized for the visualization of MR results, including forest plots, perceptual analysis plots, and scatter plots.

### 2.7 Sensitivity analyses

IVW and MR-Egger tests were employed to assess the heterogeneity. If the *p*-value was <0.05, it indicates heterogeneity in the study. IVs are considered pleiotropic if they influence outcome occurrence through factors other than exposure factors (13). Pleiotropy can lead to independence and exclusivity assumptions that do not hold. The MR-Egger intercept test can detect the pleiotropy of the data and assess the robustness of the results (14). Data were considered pleiotropic on *p*-value <0.05. Sensitivity analysis was conducted using the “leave-one-out method,” a systematic process that involves the step-by-step removal of individual SNP results. This method evaluates the combined effect of the remaining SNPs, aiming to examine whether a single SNP impacts the causal effect. Funnel plots were drawn using R language to observe whether SNPs were symmetrical and thereby determine the reliability of the findings.

### 2.8 Statistical methods and software

In this study, R 4.3.1 software^1^ and strawberry-perl 5.32.1.1 software^2^ were utilized to conduct the relationship analysis of MR. Statistical significance was determined at *p* < 0.05.

## 3 Results

### 3.1 Screening instrumental variables

**Figure 1:**
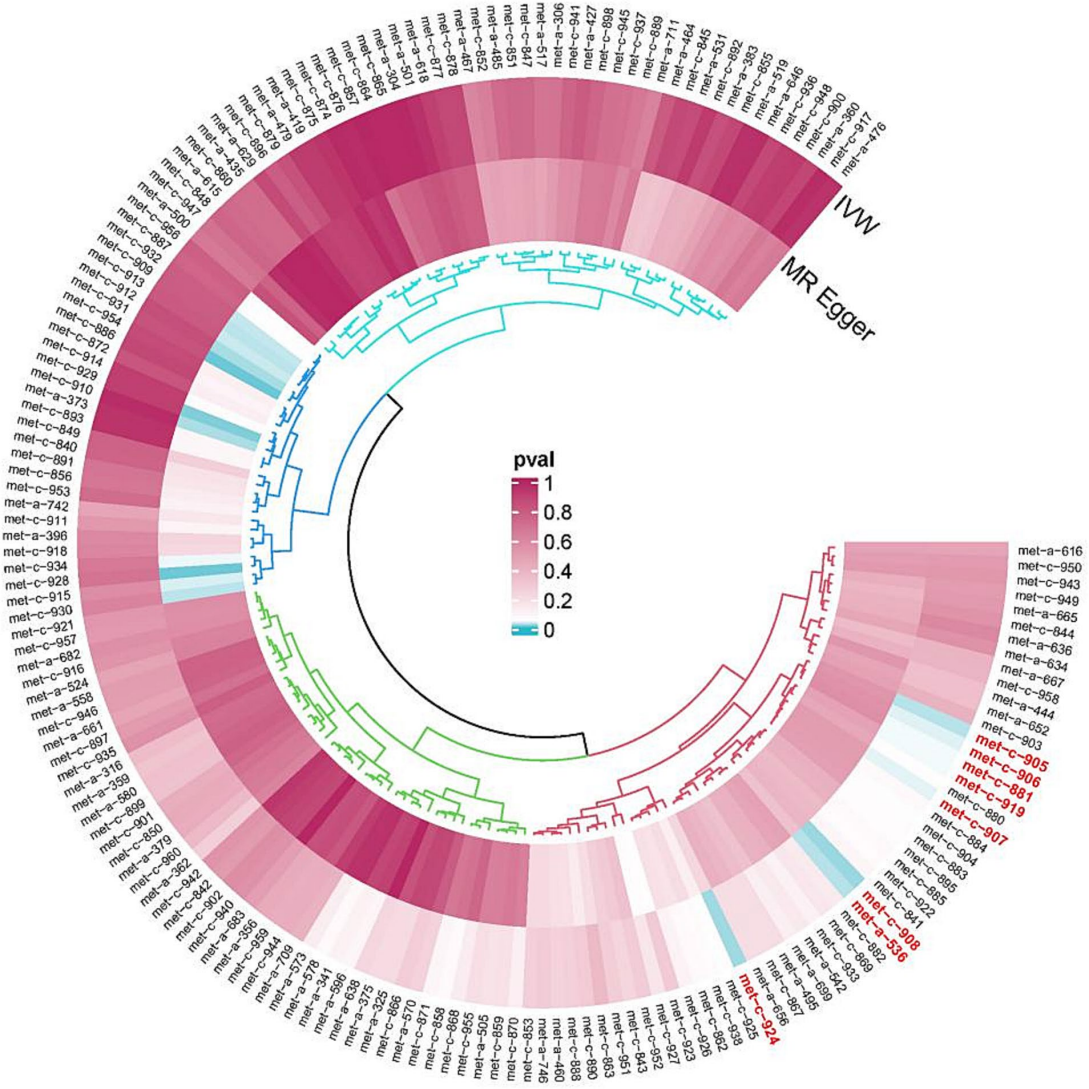
GWAS dataset of SNPs strongly associated with metabolites and ESUS.

The selection of SNPs strongly associated with exposure and outcome factors was carried out by utilizing the R packages, including “dplyr,” “gwasglue,” “tidyr,” and “VariantAnnotation”. The filtration condition was set at *p* < 5e-08. A total of 8 SNP datasets strongly linked with metabolites were identified:

met-a-536 (X-11593--O-methylascorbate*),
met-c-881 (Cholesterol esters in large very-low-density lipoprotein (VLDL)),
met-c-906 (Total lipids in medium LDL),
met-c-907 (Concentration of medium LDL particles),
met-c-908 (Phospholipids in medium LDL),
met-c-919 (Phenylalanine), and.
met-c-924 (Total cholesterol in small LDL) (Figure 1; Table 1).

**Table 1:** Metabolites related GWAS data information.

GWAS-ID	NAME	Sample	SNPs
met-a-536	X-11593--O-methylascorbate*	7,788	2,545,561
met-c-881	Cholesterol esters in large VLDL	19,273	11,820,655
met-c-905	Cholesterol esters in medium LDL	19,273	11,820,631
met-c-906	Total lipids in medium LDL	19,273	11,818,458
met-c-907	Concentration of medium LDL particles	19,273	11,818,918
met-c-908	Phospholipids in medium LDL	21,558	11,871,461
met-c-919	Phenylalanine	22,663	12,042,964
met-c-924	Total cholesterol in small LDL	21,556	11,871,461

### 3.2 Screening instrumental variables

SNPs strongly associated with exposure and outcome factors were selected employing the R packages, such as “dplyr,” “gwasglue,” “tidyr,” and “VariantAnnotation” with filtration condition of *p* < 5e-08. Among them, 6 SNPs in met-a-536 were strongly associated with ESUS; 25 SNPs in met-c-881 were strongly associated with ESUS; 25 SNPs in met-c-905 were strongly associated with ESUS; 23 SNPs in met-c-906 were strongly associated with ESUS; 23 SNPs in met-c-907 were strongly associated with ESUS; 20 SNPs in met-c-908 were strongly associated with ESUS; 4 SNPs in met-c-919 were strongly associated with ESUS; and 21 SNPs in met-c-924 were strongly associated with ESUS. *F*-value calculations and removal of confounding factors were performed for the selected SNPs by employing the R package MR. As a result, no weak IVs and confounding factors were identified (Table 2).

**Table 2:** Mitochondrial dataset tool variation scale.

GWAS-ID	Type	SNPs
met-a-536	ESUS	6
met-c-881	ESUS	25
met-c-905	ESUS	25
met-c-906	ESUS	23
met-c-907	ESUS	23
met-c-908	ESUS	20
met-c-919	ESUS	4
met-c-924	ESUS	21

### 3.3 Findings of Mendelian randomization analysis

**Figure 2:**
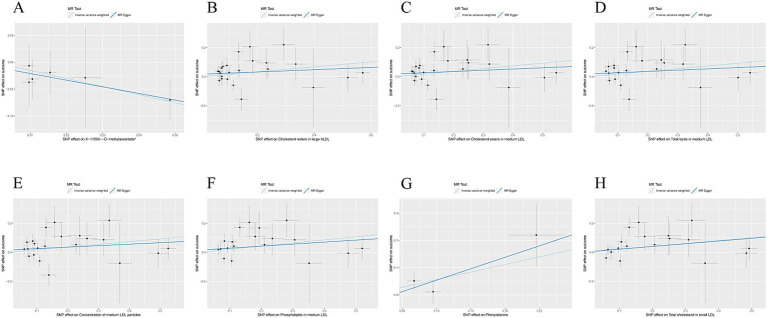
**(A)** Scatter plot of the causal relationship between X-11593--O-methylascorbate* and ESUS, mainly evaluated by IVW method; **(B)** Scatter plot of the causal relationship between Cholesterol esters in large VLDL and ESUS, mainly evaluated by IVW method; **(C)** Scatter plot of the causal relationship between Cholesterol esters in medium LDL and ESUS, mainly evaluated by IVW method; **(D)** Scatter plot of the causal relationship between Total lipids in medium LDL and ESUS, mainly evaluated by IVW method; **(E)** Scatter plot of the causal relationship between Concentration of medium LDL particles and ESUS, mainly evaluated by IVW method; **(F)** Scatter plot of the causal relationship between Phospholipids in medium LDL and ESUS, mainly evaluated by IVW method; **(G)** Scatter plot of the causal relationship between Phenylalanine and ESUS, mainly evaluated by IVW method; **(H)** Scatter plot of the causal relationship between Total cholesterol in small LDL and ESUS, mainly evaluated by IVW method.

The causal association between exposure factors and outcomes was assessed by employing MR-Egger regression and IVW analysis. Visualization of MR results was performed by utilizing the R package “TwoSampleMR,” including forest plots and scatter plots. The findings exhibited a causal relationship between X-11593--O-methylascorbate (*p* = 0.029, OR = 0.222, 95% CI: 0.058 ~ 0.858) and ESUS, indicating a protective factor. Cholesterol esters in large VLDL (*p* = 0.046, OR = 1.184, 95% CI: 1.003 ~ 1.398), cholesterol esters in medium LDL (*p* = 0.030, OR = 1.205, 95% CI: 1.018 ~ 1.426), total lipids in medium LDL (*p* = 0.041, OR = 1.204, 95% CI: 1.008 ~ 1.438), the concentration of medium LDL particles (*p* = 0.042, OR = 1.203, 95% CI: 1.006 ~ 1.437), phospholipids in medium LDL (*p* = 0.022, OR = 1.239, 95% CI: 1.031 ~ 1.489), phenylalanine (*p* = 0.046, OR = 1.749, 95% CI: 1.009 ~ 3.032), and total cholesterol in small LDL (*p* = 0.024, OR = 1.225, 95% CI: 1.028 ~ 1.461) were causally related to ESUS and were identified as risk factors (Figures 2A–G).

### 3.4 Sensitivity analyses

**Figure 3:**
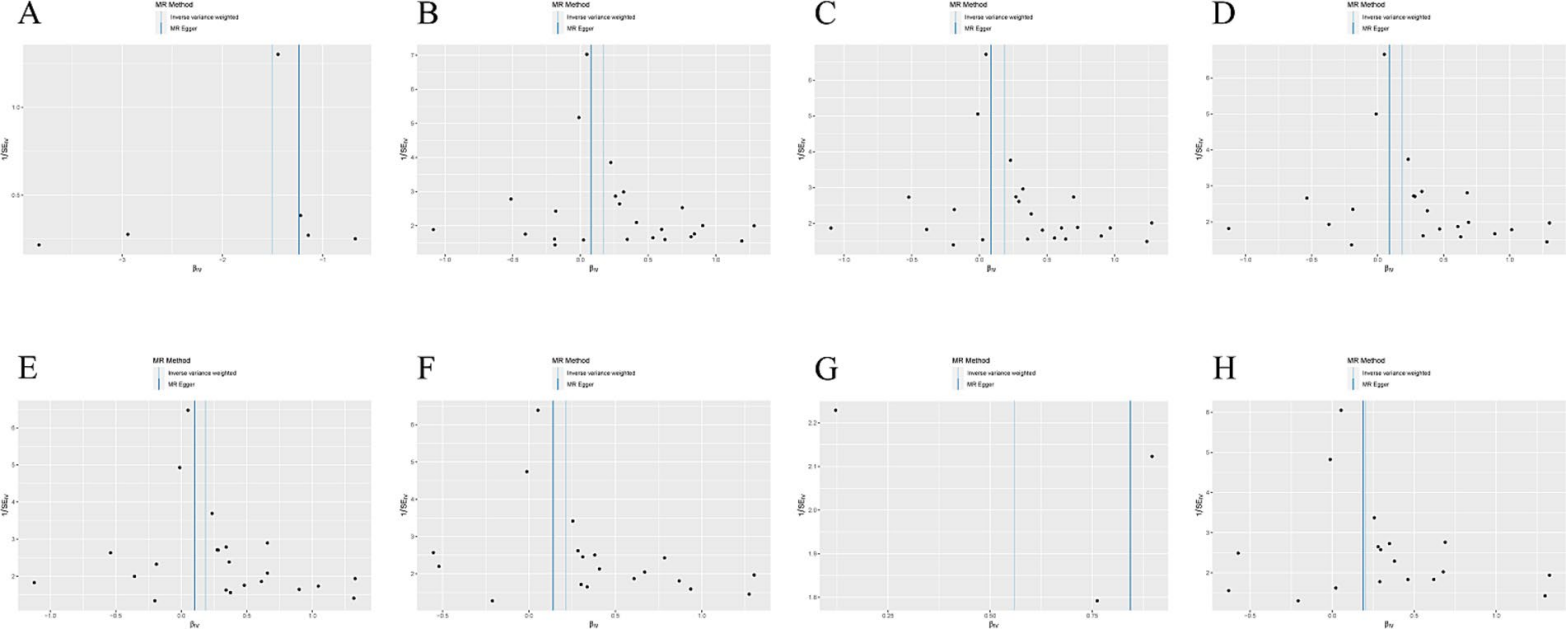
**(A)** Funnel plot of the causal relationship between X-11593--O-methylascorbate* and ESUS, mainly evaluated by IVW method; **(B)** Funnel plot of the causal relationship between Cholesterol esters in large VLDL and ESUS, mainly evaluated by IVW method; **(C)** Funnel plot of the causal relationship between Cholesterol esters in medium LDL and ESUS, mainly evaluated by IVW method; **(D)** Funnel plot of the causal relationship between Total lipids in medium LDL and ESUS, mainly evaluated by IVW method; **(E)** Funnel plot of the causal relationship between Concentration of medium LDL particles and ESUS, mainly evaluated by IVW method; **(F)** Funnel plot of the causal relationship between Phospholipids in medium LDL and ESUS, mainly evaluated by IVW method; **(G)** Funnel plot of the causal relationship between Phenylalanine and ESUS, mainly evaluated by IVW method; **(H)** Funnel plot of the causal relationship between Total cholesterol in small LDL and ESUS, mainly evaluated by IVW method.

**Figure 4:**
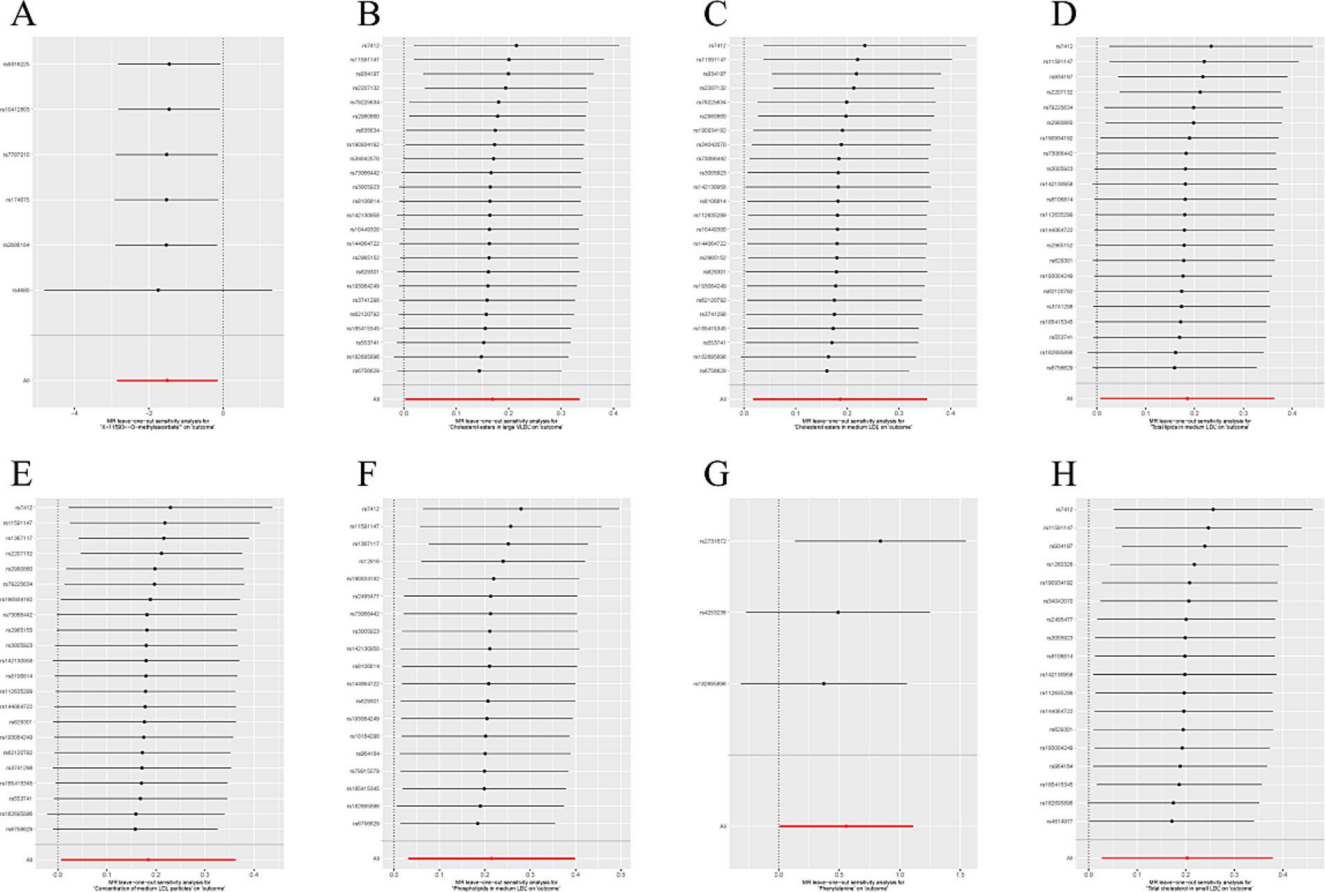
**(A)** “Leave one method” forest map of the causal relationship between X-11593--O-methylascorbate* and ESUS, mainly evaluated by IVW method; **(B)** “Leave one method” forest map of the causal relationship between Cholesterol esters in large VLDL and ESUS, mainly evaluated by IVW method; **(C)** “Leave one method” forest map of the causal relationship between Cholesterol esters in medium LDL and ESUS, mainly evaluated by IVW method; **(D)** “Leave one method” forest map of the causal relationship between Total lipids in medium LDL and ESUS, mainly evaluated by IVW method; **(E)** “Leave one method” forest map of the causal relationship between Concentration of medium LDL particles and ESUS, mainly evaluated by IVW method; **(F)** “Leave one method” forest map of the causal relationship between Phospholipids in medium LDL and ESUS, mainly evaluated by IVW method; **(G)** “Leave one method” forest map of the causal relationship between Phenylalanine and ESUS, mainly evaluated by IVW method; **(H)** “Leave one method” forest map of the causal relationship between Total cholesterol in small LDL and ESUS, mainly evaluated by IVW method.

IVW methods and MR-Egger regression were used for analyzing the heterogeneity. The results revealed that all datasets had *p* > 0.05, and no heterogeneity was observed. Egger regression orientation analysis was further used to analyze whether there was orientation level multidirectionality. The results showed that all datasets had *p* > 0.05, and no orientation-level multidirectionality was observed (Table 3). The funnel plot showed that SNPs were symmetrically distributed and the results obtained were relatively stable (Figure 3). The leave-one-out method was used to eliminate SNPs one by one and observe any changes in the effect values. The results indicated that no strong influential SNP loci were identified (Figure 4).

**Table 3:** Heterogeneity analysis.

GWAS-ID	Type	Heterogeneity tests		Directional horizontal pleiotropy tests
		MR Egger	IVW	
met-a-536	ESUS	0.983	0.993	0.780
met-c-881	ESUS	0.146	0.151	0.401
met-c-905	ESUS	0.158	0.160	0.365
met-c-906	ESUS	0.105	0.110	0.419
met-c-907	ESUS	0.109	0.122	0.499
met-c-908	ESUS	0.181	0.209	0.560
met-c-919	ESUS	0.240	0.450	0.762
met-c-924	ESUS	0.277	0.337	0.904

## 4 Discussion

In this study, public large-sample GWAS data were employed for analyzing the potential causal association between metabolites and ESUS using R language. X-11593--O-methylascorbate* (IWV, *p* = 0.029, OR = 0.222, 95% CI: 0.058 ~ 0.858) exhibited a causal relation with ESUS and was identified as a protective factor. Cholesterol esters in large VLDL (IWV, *p* = 0.046, OR = 1.184, 95% CI: 1.003 ~ 1.398), cholesterol esters in medium LDL (IWV, *p* = 0.030, OR = 1.205, 95% CI: 1.018 ~ 1.426), total lipids in medium LDL (IWV, *p* = 0.041, OR = 1.204, 95% CI: 1.008 ~ 1.438), concentration of medium LDL (IWV, *p* = 0.042, OR = 1.203, 95% CI: 1.006 ~ 1.437), phospholipids in medium LDL (IWV, *p* = 0.022, OR = 1.239, OR = 1.239, 95% CI: 1.031 ~ 1.489), phenylalanine (IWV, *p* = 0.046, OR = 1.749, 95% CI: 1.009 ~ 3.032), and total cholesterol in small LDL (IWV, *p* = 0.024, OR = 1.225, 95% CI: 1.028 ~ 1.461) were causally associated with ESUS and were identified as risk factors.

Stroke represents a serious condition that is categorized into ischemic and hemorrhagic strokes. Among them, ischemic stroke without a clear cause is called cryptogenic stroke, with ESUS being one of its subtypes (15, 16). Alterations in metabolite levels are regarded as the primary response of biological systems to various biological events. They can amplify small changes in genes and proteins and, more directly, precisely, and sensitively, reflect the physiological and pathological conditions of the body. Consequently, metabolites are associated with numerous human diseases (17). Recent studies indicate that metabolites are related to stroke and play crucial roles in its onset and progression. Inhibition of metabolite levels has been shown to delay the occurrence and development of ESUS.

This study presents several advantages. Firstly, it utilized large sample GWAS data, ensuring the reliability of the results. Secondly, SNPs were carefully screened through association analysis, LD analysis, and the removal of weak instrumental variables and confounders, effectively mitigating potential bias (18, 19). Thirdly, the symmetrical distribution of SNPs was confirmed by funnel plots, and the relatively stable results were supported by pleiotropy and leave-one-out tests. Lastly, the exploration of the molecular mechanisms by investigating the causal association between metabolites and ESUS, with metabolites as exposure factors, holds significant clinical relevance (20).

This study has several limitations. Although MR-Egger regression and IVW methods indicated no significant heterogeneity between the analyses (*p* > 0.05), potential sources of heterogeneity may arise from variations in instrumental variables (IVs), including differences in analytical platforms, experimental designs, and population characteristics, necessitating a more detailed exploration of these sources to enhance the validity of the findings. Additionally, the GWAS data are predominantly derived from European populations, resulting in a lack of representation from African and Asian cohorts; thus, future research should include diverse populations for a more comprehensive understanding of the associations examined. The number of IVs for SNPs within each dataset is relatively limited, indicating a need for larger sample sizes to identify additional SNPs as IVs. While this research utilized Mendelian Randomization (MR) to analyze the causal relationship between metabolites and ESUS, no mechanistic studies were conducted to elucidate the underlying biological processes, which could provide valuable insights. Furthermore, the findings may be influenced by residual confounding factors not fully accounted for in the analyses, and it is essential to consider these impacts in future investigations. Lastly, the analytical methods employed may have limitations in detecting all possible causal relationships, warranting the exploration of alternative methodologies, such as sensitivity analyses or different statistical approaches, to validate the robustness of the results.

## 5 Conclusion

This research evaluated the potential causal effect of metabolites on embolic stroke of undetermined source (ESUS) through Mendelian Randomization (MR) analysis. The findings revealed that X-11593-O-methylascorbate is causally related to ESUS, functioning as a protective factor. Conversely, several metabolites, including cholesterol esters in large very-low-density lipoprotein (VLDL), cholesterol esters in medium low-density lipoprotein (LDL), total lipids in medium LDL, concentration of medium LDL particles, phospholipids in medium LDL, phenylalanine, and total cholesterol in small LDL, were identified as risk factors associated with ESUS.

While these outcomes provide a theoretical basis for further investigations into the dynamics of metabolite levels during ESUS, it is crucial to explore the underlying biological mechanisms connecting these metabolites to ESUS (21). Future studies should incorporate experimental approaches to better understand how specific metabolites influence the pathophysiology of ESUS (22, 23). For example, *in vitro* studies could be conducted to investigate the effects of these metabolites on endothelial function, coagulation pathways, and inflammatory responses, which are critical in the development of ESUS. Understanding these mechanisms would not only clarify the causal relationships identified but also inform clinical interventions aimed at modifying metabolite levels to reduce the risk of ESUS. In addition to exploring biological mechanisms, it is essential to strengthen the clinical application prospects of the findings (24, 25). The study suggests that managing metabolite levels may help reduce the incidence of ESUS; however, the discussion lacks a thorough exploration of how these findings can be translated into clinical practice. Specifically, there is a need to elaborate on potential directions for drug development and personalized treatment strategies based on the identified metabolites (26).

In summary, while this study lays a solid foundation for understanding the causal relationships between metabolites and ESUS, future research should prioritize elucidating the underlying biological mechanisms and translating these findings into actionable clinical strategies. By doing so, it may be possible to not only mitigate the risk of ESUS but also pave the way for innovative therapeutic approaches that harness the power of metabolite modulation in clinical practice.

## Data Availability

The datasets presented in this study can be found in online repositories. The names of the repository/repositories and accession number(s) can be found in the article/supplementary material.

## Footnotes

^1^ https://cloud.r-project.org/

^2^ https://strawberryperl.com/
